# Does Lymph Node Dissection Impact Adjuvant Treatment or Survival Outcomes in High-Risk Endometrial Cancers?

**DOI:** 10.7759/cureus.24710

**Published:** 2022-05-03

**Authors:** Breana L Hill, Kelsey Goon, Joellen Fresia, Jeanelle Sheeder, Rebecca J Wolsky, Jill Alldredge

**Affiliations:** 1 Obstetrics and Gynecology, University of Colorado Hospital School of Medicine, Aurora, USA; 2 Obstetrics and Gynecology, University of Colorado School of Medicine, Aurora, USA; 3 Pathology, University of Colorado Hospital School of Medicine, Aurora, USA; 4 Obstetrics and Gynecology/Gynecologic Oncology, University of Colorado Hospital School of Medicine, Aurora, USA

**Keywords:** progression-free survival, adjuvant therapy, overall survival, sentinel lymph nodes, high-risk endometrial cancers

## Abstract

Objectives

Lymphadenectomy does not improve overall survival outcomes in patients with low-risk endometrial cancers. Sentinel node mapping has a high detection rate and accuracy; however, its prognostic implications have not been well explored. We evaluated the overall survival and therapies received by patients undergoing varied lymph node dissection approaches for high-risk endometrial cancers.

Methods

Retrospective review of grade 3 endometrioid and high-grade non-endometrioid cancers at one institution over ten years. Patients who received neoadjuvant therapy and/or debulking of only grossly abnormal lymph nodes were excluded. Data was abstracted from electronic medical records. Chi-squared tests and survival analyses were used to compare groups.

Results

One hundred and fifty-three patients with grade 3 endometrioid, serous, clear cell, carcinosarcoma, or mixed high-grade on final pathology were identified; 16 had no lymph node dissection, 26 had sentinel lymph nodes, and 111 had complete lymph node dissection. Patients with open surgery were more likely to have complete nodes than sentinel nodes when compared to a minimally invasive approach (p<0.001). Sentinel nodal dissection significantly impacted the utilization of, or modality choice, in adjuvant therapy (p=0.051). Recurrence-free survival and cancer-specific overall survival were not significantly different across the three nodal-assessment groups.

Conclusions

Sentinel lymph node dissection in high-risk endometrial cancers led to no significant differences in recurrence-free survival or cancer-specific overall survival. While limited by sample size and its retrospective nature, results from this single-institution study are hypothesis-generating and prompt consideration of non-inferiority trials. Performing the least invasive surgery possibly can lead to fewer complications while maintaining overall survival outcomes.

## Introduction

Uterine cancer is the most common gynecologic malignancy in developed countries. While adenocarcinoma is the most common histologic type of uterine cancer, type II neoplasms (including serous, clear cell, mucinous, squamous, transitional cell, mesonephric, and undifferentiated) comprise 10-20% of endometrial carcinomas and account for 40% of deaths from the disease [1]. Both serous carcinomas and clear cell tumors have a higher propensity for lymphovascular invasion and intraperitoneal and extra-abdominal spread than their endometrioid counterparts. Additionally, they are more likely to be diagnosed at an advanced stage [2]. The five-year overall survival rate for patients has been reported as 18% to 27%, likely secondary to the extrauterine spread in 60-70% of the patients at the time of diagnosis [3].

According to Federacion International Gynecologica Oncology (FIGO) and National Comprehensive Cancer Network (NCCN) guidelines, an endometrial biopsy with non-endometrioid histology warrants consideration of cancer antigen 125 (CA-125) testing and imaging, followed by a hysterectomy, bilateral salpingo-oophorectomy, and surgical staging with pelvic lymphadenectomy and consideration of periaortic lymphadenectomy, cytology, omentectomy, and peritoneal biopsies, with an effort to debulk gross disease. Depending on the stage of the disease, this is followed by observation, chemotherapy, radiation, or a combination of the latter two. Recent data confirms the utility of sentinel lymph nodes as a diagnostic test within high-risk uterine cancers, with high sensitivity, specificity, and negative predictive value [4]. With the advent of this technique, the Society of Gynecologic Oncology (SGO) supports it as it allows accurate staging and decrease in morbidity of surgery, such as lymphedema, when used within the framework of an algorithm [5]. Further data suggests that sentinel nodes rather than full lymphadenectomy do not impact progression-free survival (PFS) or overall survival (OS) [6]. Given the range of these guidelines for lymph node dissection in patients with high-grade, non-endometrioid endometrial cancers, it is important for the gynecologic oncologist to weigh the risks of lymphadenectomy.

We specifically aim to assess whether the use of sentinel nodes as compared to complete or absent nodal dissection altered adjuvant treatment choices in those women with a diagnosis of high-grade, non-endometrioid endometrial cancer. Furthermore, we sought to corroborate the existing data suggesting that PFS and OS are not altered by the use of sentinel nodes. We hypothesized similar rates of progression-free and overall survival across the treatment groups and that the use of sentinel nodal assessment does not impact adjuvant therapy decisions.

This article was previously presented as a poster at the 2021 Western Association of Gynecologic Oncologists meeting on June 16, 2021.

## Materials and methods

Colorado Multiple Institutional Review Board (COMIRB) application was submitted prior to study initiation. Under the title "Non-endometrioid endometrial cancers: lymph node dissection and survival outcomes" and ID number APP001-1, this study was determined to be exempt.

Women aged 18 to 99 who underwent surgery or had pathology reviewed at the University of Colorado Hospital and had grade 3 endometrioid or high-grade, non-endometrioid endometrial cancer on final pathology between January 1, 2010, and January 1, 2020, were included (n=236). Patients who received neoadjuvant therapy and/or debulking of only grossly abnormal lymph nodes were excluded (n=83). Since 2016 at this institution, the sentinel lymph node processing protocol in pathology includes two hematoxylin and eosin-stained levels and two levels stained with pan-keratin (AE1/3 and CAM5.2). Medical records, operative, and pathology reports were reviewed for patient and disease characteristics.

Descriptive statistics were used to report the rate of patients with a diagnosis of high-grade non-endometrioid endometrial cancers receiving sentinel lymph node dissection and those receiving complete lymph node dissection. Patients with sentinel lymph node dissection were compared to those who received complete lymph node dissection or no lymph node dissection using chi-square for categorical variables, Student's t-test was used for continuous variables that are normally distributed, and nonparametric tests were used for continuous variables that are not normally distributed. A p-value ≤0.05 was considered statistically significant.

## Results

A total of 153 patients met the inclusion criteria. Of those, 16 had no lymph node dissection (10.4%), 26 had sentinel lymph node dissection (17.0%), and 111 had a complete lymph node dissection (72.5%). Included patients showed no difference in age, ethnicity, stage of disease, or histology (Table 1). Most patients had stage I disease (67%) and serous histology (46%). Those who received open surgery were more likely to undergo complete pelvic nodal dissection as compared to sentinel nodes or no nodal dissection (p<0.0001). Additionally, across the three nodal-dissection groups, there were no differences in cancer recurrence, site of cancer recurrence, or death from cancer.

**Table 1:** Patient, tumor characteristics, and nodal dissection approach

Patient and tumor characteristics	No nodes N (%)	Sentinel nodes N (%)	Complete nodes N (%)	p
Mean age (years)	68.7	65.1	64.0	0.42
Ethnicity				
White Hispanic	4 (25%)	0 (0%)	7 (6.3%)	0.09
White non-Hispanic	9 (56.3%)	24 (92.3%)	94 (84.7%)	
Black	1 (6.3%)	1 (3.8%)	5 (4.5%)	
Asian	0 (0%)	0 (0%)	1 (0.9%)	
Other	2 (12.5%)	1 (3.8%)	4 (3.6%)	
Surgical approach				
Laparoscopic	0 (0%)	11 (42.3%)	13 (11.7%)	<0.001
Robotic	9 (56.3%)	9 (34.6%)	33 (29.7%)	
Open	7 (43.8%)	6 (23.1%)	65 (58.6%)	
Stage				
IA	9 (56.3%)	10 (38.5%)	51 (45.9%)	0.80
IB	6 (37.5%)	7 (26.9%)	19 (17.1%)	
II	1 (6.3%)	1 (3.8%)	7 (6.3%)	
IIIA	0 (0%)	3 (11.5%)	11 (9.9%)	
IIIB	0 (0%)	0 (0%)	1 (0.9%)	
IIIC1	0 (0%)	2 (7.7%)	6 (5.4%)	
IIIC2	0 (0%)	1 (3.8%)	10 (9.0%)	
IVA	0 (0%)	0 (0%)	1 (0.9%)	
IVB	0 (0%)	2 (7.7%)	5 (4.5%)	
Histology				
Serous	5 (31.3%)	13 (50.0%)	45 (40.5%)	0.26
Grade 3 endometrioid	2 (12.5%)	6 (23.1%)	30 (27%)	
Clear cell	0 (0%)	2 (7.7%)	4 (3.6%)	
Carcinosarcoma	4 (25%)	3 (11.5%)	20 (18.0%)	
Mixed high-grade histology	5 (31.3%)	2 (7.7%)	12 (10.8%)	
Outcomes				
Cancer recurrence (y/n)	5	3	32	0.17
Vaginal	4	1	10	0.07
Pelvic node	0	1	3	0.75
Para-aortic node	0	0	6	0.31
Distant	4	2	21	0.29
Death from cancer	2	1	24	0.22

Of the nodes resected, pathology showed that no nodal metastases were the most common between the two nodal dissection groups (Table 2). More macrometastases were identified in the complete nodal group than in the sentinel nodal group (p=0.03).

**Table 2:** Pathology of nodal resection

Pathology	Sentinel nodes N (%)	Complete nodes N (%)	p
No nodal metastases	21(80.8%)	93 (83.8%)	0.03
Isolated tumor cells	1 (3.8%)	0 (0%)	
Micrometastases	1 (3.8%)	0 (0%)	
Macrometastases	3 (11.5%)	18 (16.2%)	

Adjuvant therapy was divided into four groupings: surgery alone, chemotherapy only, chemotherapy with radiation therapy, and radiation alone (Table 3). Across the three nodal-dissection groups, surgery alone was significantly more common in the complete nodal dissection group. Six cycles of carboplatin/paclitaxel with trastuzumab plus vaginal brachytherapy were most likely to be received by the no-nodal-assessment group (n=1).

**Table 3:** Adjuvant treatment utilization by nodal assessment C/T - carboplatin and paclitaxel; VBT - vaginal brachytherapy; EBRT - external beam radiation therapy

Adjuvant therapy	No nodes N	Sentinel nodes N	Complete nodes N	p
No adjuvant therapy				
Surgery alone	7	4	14	0.007
Chemotherapy only				
6 cycles of C/T	1	5	19	0.50
6 cycles of C/T + trastuzumab	0	1	0	0.09
Fewer than 6 cycles C/T	2	2	10	0.87
Chemotherapy + radiation				
6 cycles C/T + VBT	3	7	41	0.26
6 cycles C/T + trastuzumab + VBT	1	0	0	0.013
C/T-EBRT-C/T sandwich	0	4	12	0.28
Radiation only				
EBRT alone	1	0	6	0.47
VBT alone	0	2	9	0.50
EBRT+VBT	2	0	3	0.07
Other	0	1	0	0.09

Recurrence-free survival and cancer-specific overall survival were not significantly different across the three nodal assessment groups. As shown in Table 4, recurrence-free survival (RFS) was 61.2 months, 70.3 months, and 86.9 months for no-nodal assessment, sentinel node dissection, and complete nodal dissection, respectively. Additionally, overall survival was not statistically different between the three groups.

**Table 4:** Patient outcomes by nodal assessment

Outcomes (months)	No nodes, months (95% CI)	Sentinel nodes, months (95% CI)	Complete nodes, months (95% CI)	p
Recurrence-free survival	61.2 (33.3-89.2)	70.3 (35.2-103.4)	86.9 (74.0-97.5)	0.630
Cancer-specific overall survival	92.3 (78.4-106.2)	89.7 (59.1-102.3)	96.4 (85.9-106.9)	0.476

**Figure 1:**
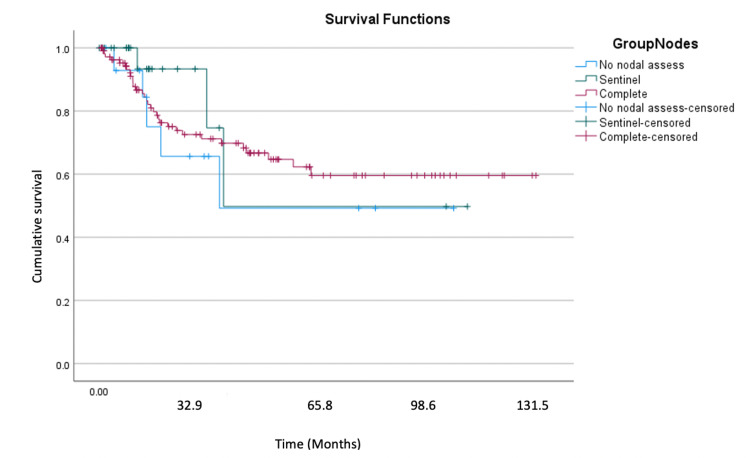
Kaplan-Meier curve for recurrence-free survival

**Figure 2:**
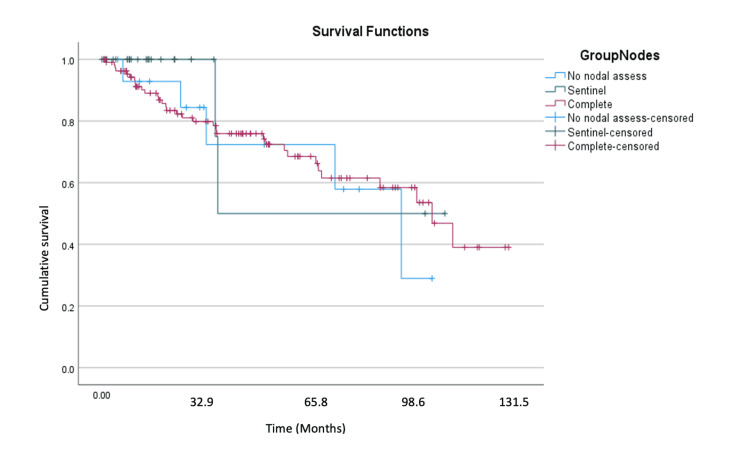
Kaplan-Meier curve for cancer-specific overall survival

Figures 1-2 show the Kaplan-Meier curves for recurrence-free and cancer-specific overall survival. At the end of the study period, we found that 50% of the no nodal assessment and sentinel nodal dissection groups were without recurrence, while this was 60% in the complete nodal dissection group. At the end of the study period, the cumulative survival of the sentinel node group was the highest, at 50%. The cumulative survival of the no nodal assessment group was the lowest at 30%. The complete nodal dissection cumulative survival at 131.5 months was 40%.

## Discussion

In our retrospective study of 153 patients with high-grade uterine cancer who underwent no nodal assessment, sentinel nodal dissection, or complete nodal dissection during the initial surgery, we found no difference in progression-free or overall survival. There was a statistically significant difference in adjuvant therapy received, with the complete nodal dissection group being more likely to receive surgery alone and the no nodal assessment group more likely to receive chemotherapy with trastuzumab and vaginal brachytherapy than the other two groups. However, interpretations of this outcome may be limited by the small sample size of the patients with sentinel nodal dissection. We anticipate that the difference in adjuvant therapy would decrease with larger studies. If indeed such a difference in practice exists, it is possible that in those women with complete nodal dissection, providers extrapolate the diagnostic impact of their dissection as a therapeutic benefit and may feel that the utility of adjuvant therapy is outweighed by toxicity. This analysis cannot speak to the rationale for a provider's nodal dissection choices; thus, there may be patient or disease factors impacting the decision to exclude nodal assessment that is not captured here. In situations in which nodal assessment is not performed at all, providers may be inclined to pursue broader and more aggressive adjuvant therapy combining radiotherapy and chemotherapy modalities when the nodal status is unknown, and stage III disease cannot be definitively excluded.

The route of surgery was impacted by the nodal dissection method. Those who had open surgery were more likely to have complete nodal dissection, while the sentinel nodal dissection and no-nodal assessment groups were more commonly approached minimally invasively. Given technologic limitations for applying sentinel nodal algorithms in open surgery, this finding is not unsurprising, and expansion of uptake of sentinel nodal dissection during open surgery would not be expected until non-laparoscopic infrared-lighting technology becomes more widely available.

Studies have shown that lymphadenectomy does not improve overall survival outcomes in patients with type I, low-risk endometrial cancers, but limited data exist investigating its utility in the treatment of type II, high-grade, high-risk endometrial cancers [7]. The importance of nodal assessment in staging and outcomes was established by Eggemann et al., who conducted a multicenter, retrospective study of 1,502 patients with endometrial cancer treated with no lymphadenectomy, pelvic lymphadenectomy, and pelvic/para-aortic lymphadenectomy. Patients were divided into groups with a low, intermediate, and high risk of recurrence, with overall survival as the primary outcome. They concluded that combined pelvic and para-aortic lymphadenectomy significantly reduced the mortality risk in patients with an intermediate and high risk of recurrence compared with no lymphadenectomy. The limitations of the study were that disease-free survival and rate of recurrence were not investigated, and exact FIGO staging subgroups, as well as patient comorbidities (which may have confounded the results), were not studied [8]. Furthermore, a portion of the cases studied was performed before the implementation of sentinel nodal dissection.

Lymphadenectomy is not without risk. Lymphadenectomy has been shown to increase the median operating time by 60 minutes, increase surgery-related systemic morbidity, and increase the duration of inpatient hospital stay [9]. Women with lymphadenectomy also have a higher risk of lymphoedema and formation of lymphocyst and experience larger blood loss and increased need for blood transfusion [10]. Prior studies have suggested that sentinel lymph node dissection has the performance capabilities to replace lymphadenectomy. The Sentinel Lymph Node Biopsy vs. Lymphadenectomy for Intermediate- and High-Grade Endometrial Cancer Staging (SENTOR) study also looked at high-grade endometrial cancer and identified 89% of patients with a node-positive disease with sentinel lymph node biopsy [11]. Other studies [12-14] found similar sensitivities (96-98%) and negative predictive values (99%) but focused on grade 1 or 2 endometrioid endometrial cancers only. While our study did not look at the diagnostic accuracy of sentinel lymph node dissection at our institution, we did find that it did not change PFS or OS.

Determining the Sensitivity of Sentinel Lymph Nodes Identified With Robotic Fluorescence Imaging (FIRES) trial showed that sentinel lymph nodes contained metastatic disease more often than non-sentinel lymph nodes, and 54% of patients with positive sentinel lymph nodes had a small-volume disease that would have been missed without ultrastaging [13]. Other studies suggest that sentinel lymph node dissection increases the detection of micrometastases and isolated tumor cells by 4% to 25% [15-18], which may or may not have an impact on adjuvant therapy. In light of Randomized Trial of Radiation Therapy With or Without Chemotherapy for Endometrial Cancer (PORTEC-3), Gynecologic Oncology Group(GOG) 249, and GOG 258, adjuvant therapy options for high-risk or advanced endometrial cancers are widely variable, and the standard of care remains unclear, leaving much to provider discretion [19-21]. The large number of adjuvant therapy modalities we identified in our retrospective review further elucidates that there is not a one-size-fits-all approach.

Limitations of our study include the retrospective nature and the limited number of patients who received sentinel lymph node dissection at our institution in this 10-year period. We anticipate an increase in the utilization of sentinel nodal dissection in the coming years as additional studies confirm its performance capabilities and confirm our results of lack of impact on PFS or OS. With an increasing sample size, we predict that the differences detected in adjuvant therapy choices based on the nodal approach would become non-significant. Strengths of our study involve the inclusion of high-risk endometrial cancers of all stages, evaluation of adjuvant therapy received in relation to nodal assessment, and evaluation of these variables in the context of progression-free and overall survival outcomes.

## Conclusions

Sentinel nodal dissection appears to be the most effective way to identify small volume metastases, and with its decreased surgical risk and lack of impact on OS or PFS, it is reasonable to conclude that sentinel nodal dissection should be adopted into the routine practice of grade 3 endometrioid and high-risk non-endometrioid uterine cancers. Complete lymphadenectomy for patients with high-risk endometrial cancers does not appear to impact progression-free or overall survival outcomes. Women with open surgery were more likely to have a complete nodal dissection, supporting that a minimally invasive technique should be utilized when patient and uterine factors allow. While we could not conclude that there was not a difference in adjuvant therapy treatment received by each of the nodal groups, differences may have been a result of the limited study sample, and further investigation is warranted. Our findings suggest that it is appropriate to continue to perform sentinel nodal dissection in appropriately selected patients with grade 3 endometrioid or high-risk non-endometrioid type endometrial cancers without concern for impacting PFS or OS.
